# Gaining muscle mass in COPD: a work in progress

**DOI:** 10.1183/23120541.00336-2023

**Published:** 2023-07-31

**Authors:** Hamish J.C. McAuley, Matthew Maddocks

**Affiliations:** 1 Institute of Lung Health, University of Leicester, Leicester, UK; 2 Cicely Saunders Institute of Palliative Care, Policy and Rehabilitation, King's College London, London, UK

## Abstract

Despite its definition as a disease of the airways, chronic obstructive pulmonary disease (COPD) is widely recognised as a systemic illness that affects multiple organs and systems [1]. In particular, the loss of skeletal muscle and function among people living with COPD has been well characterised with regards to its prevalence, mechanisms, and impact on quality of life and prognosis [2–4].

## Tweetable abstract

**Interventions to improve muscle mass in COPD remain limited to exercise and nutrition. As novel drugs show promise, there should be focus on identifying target subgroups of patients, strategies to combine interventions and optimise outcome measures.** https://bit.ly/3WI5Sjg

Despite its definition as a disease of the airways, chronic obstructive pulmonary disease (COPD) is widely recognised as a systemic illness that affects multiple organs and systems [1]. In particular, the loss of skeletal muscle and function among people living with COPD has been well characterised with regards to its prevalence, mechanisms, and impact on quality of life and prognosis [2–4].

Indeed, while work continues to better understand muscle dysfunction in COPD, its management with pulmonary rehabilitation (in one form or another) is recognised and endorsed by clinical guidelines across many countries.

A systematic review and meta-analysis in *ERJ Open Research* by Jenkins *et al*. [5] summarised the current evidence around treatments for low muscle mass in this population. The review identified three main intervention strategies that have been investigated for their potential to improve muscle mass in COPD as exercise (including resistance) training, nutritional supplementation and anabolic steroids. In addition, more limited data on the assessment of pharmacological interventions including hormonal therapies, angiotensin-converting enzyme inhibitors and antibody therapy were reviewed. While the article highlights common challenges in pooling data in this field, the results suggest that exercise training, nutritional supplementation and anabolic steroids alone each lead to gains in muscle mass, with combinations of exercise and nutrition showing promise of incremental benefit. Two striking similarities hold between the interventions on this list; firstly, they are not novel, with exercise and nutrition interventions likely dating to before modern medicine, and anabolic steroids being first invented nearly a century ago [6]. Secondly, the real-world utility of the interventions has been limited by treatment tolerability and/or side-effects, particularly among those with progressive illness [7, 8].

Fortunately, additional novel therapies are being developed, though few have been tested or reported for their efficacy among patients with low muscle mass or sarcopenia [9]. For people living with COPD specifically, two drugs have so far reached phase II evaluation in placebo-controlled studies. A study of bimagrumab, a monoclonal antibody blocking the activin type II receptor, in adults with COPD and low total skeletal muscle mass showed a 5.0% increase in thigh muscle volume over 24 weeks of treatment [10]. The selective androgen receptor modulator GSK2881078 improved muscle mass over 13 weeks of treatment among COPD patients with evidence of reduced performance in a five sit-to-stand test, though strength only improved in male participants [11].

These drug studies and the review Jenkins *et al*. [5] highlight important considerations for research into therapies to (re)build muscle mass among patients with COPD or other chronic diseases. First, the choice of participant group needs careful consideration; not all patients with COPD have muscle wasting and it does not always translate to a clinically relevant consequence. In an attempt to target intervention testing to groups with greater potential to benefit, investigators have used measures of muscle performance (five sit-to-stand test) [11] or total skeletal muscle mass (measured as either low body mass index or appendicular skeletal muscle mass index) to enrich study samples [10]. Jenkins *et al*. [5] note that subanalyses of studies assessing the effect of anabolic steroids among “depleted” *versus* “non-depleted” COPD patients suggest a clear divergence in effects, with greatest benefit among those who were malnourished, underweight or sarcopenic. The inconsistency in such assessment and definition of low muscle mass across the literature is problematic and inhibits like-for-like comparison. Nonetheless, more uniform assessment should emerge with consensus-defined and practical measures of sarcopenia from the European Working Group on Sarcopenia in Older People [12].

In addition to selecting patients with appropriate phenotypes, consideration should be made to the timing of interventions. It is accepted that muscle loss among older people, and in particular those with COPD, occurs as a gradual progression and it has been hypothesised that this process is accelerated in some individuals compared to others [12]. On this background, acute events such as exacerbations and/or hospitalisations lead to step changes in the loss of muscle mass, with variable and clinically relevant heterogeneity in recovery [13]. These events offer further potential to target interventions, for example to individuals with stable COPD demonstrating a period of accelerated muscle loss or to those recently hospitalised and at increased risk of poor/slow recovery.

A key finding from the review by Jenkins *et al*. [5] was the observation that combining interventions such as nutritional supplementation with exercise showed promise towards an additive effect on muscle mass. This concept may be even more relevant to explore for novel pharmacological interventions where risk profiles may inhibit long term use. Combining these with interventions that can be continued long-term, like exercise, may lead to sustained benefits. Furthermore, the nature of muscle as a “metabolically costly” tissue may mean the co-administration of muscle-promoting drugs and exercise training helps catalyse effective and relevant muscle growth. The interaction between anabolic medicines and pulmonary rehabilitation is largely unexplored but exciting.

Another final consideration highlighted by this work is the choice of outcome measures. Multiple modalities and measures of muscle mass are available, including dual-energy X-ray absorptiometry, bioelectrical impedance analysis, computed tomography, magnetic resonance imaging and ultrasound, with different options of segmental or whole-body assessment. Each has its advantages and shortcomings, and investigators are balancing cost and precision with availability and practicalities. Further complicating this choice is the multiple means of assessing muscle strength, which are similarly (if not more) broad and varied. While there are risks associated with any drive to homogenise these assessments, not least that suboptimal assessment modalities become embedded practice, a consensus on a minimum common set of measures should be a research priority to ensure future comparability as highlighted by Jenkins *et al*. [5]. Beyond phase II studies, investigators and regulators will need to agree upon suitable clinically relevant outcome measures for use in drug licensing applications. While most drug approvals for COPD have been on the basis of effects on spirometry or exacerbation frequency, these are highly unlikely to be relevant for those targeting muscle loss. Improvements in function, symptom experience and health-related quality of life are more likely candidate outcomes. The St George's Respiratory Questionnaire has been acknowledged as a co-primary endpoint for COPD studies in Europe and the USA [14]. Such measures may be best suited to capture the multiple domains of improvement that are sought [15], however examples of their use as primary endpoints in phase III drug trials are limited.

In summary, research into interventions to address low muscle mass and its consequences among people living with COPD has progressed substantially over the past decades, although the translation of findings into clinical care is largely confined to pulmonary rehabilitation. Given the progress to date, efforts should now focus on the identification of subgroups and biomarkers to identify individuals most likely to benefit; gaining consistency in the use of relevant endpoints; and finally, to examine how combinations of intervention may be used synergistically and to best effect. Given the paucity of new treatments in COPD that improve physical function and health-related quality of life, the clinical need around muscle dysfunction remains high.
